# Citicoline Treatment in Acute Ischemic Stroke: A Randomized, Single-Blind TMS Study

**DOI:** 10.3389/fneur.2022.915362

**Published:** 2022-07-13

**Authors:** Enrico Premi, Valentina Cantoni, Alberto Benussi, Nicola Gilberti, Veronica Vergani, Ilenia Delrio, Massimo Gamba, Raffaella Spezi, Angelo Costa, Alessandro Padovani, Barbara Borroni, Mauro Magoni

**Affiliations:** ^1^Stroke Unit, Azienda Socio Sanitaria Territoriale Spedali Civili, Brescia, Italy; ^2^Neurology Unit, Department of Clinical and Experimental Sciences, University of Brescia, Brescia, Italy; ^3^Department of Molecular and Translational Medicine, University of Brescia, Brescia, Italy; ^4^Neurology Unit, Department of Neurological and Vision Sciences, ASST Spedali Civili, Brescia, Italy

**Keywords:** stroke, transcranial magnetic stimulation, short-latency afferent inhibition (SAI), citicoline, cholinergic system (CS)

## Abstract

**Background:**

Recent research on animal models of ischemic stroke supports the idea that pharmacological treatment potentially enhancing intrinsic brain plasticity could modulate acute brain damage, with improved functional recovery. One of these new drugs is citicoline, which could provide neurovascular protection and repair effects.

**Objectives:**

The objective of this randomized, single-blind experimental study was to evaluate whether the treatment with Rischiaril^®^ Forte was able to restore intracortical excitability measures, evaluated through transcranial magnetic stimulation (TMS) protocols, in patients with acute ischemic stroke.

**Methods:**

Patients with acute ischemic stroke were recruited and assigned to an eight-week therapy of standard treatment (control group - CG) or CDP-choline (Rischiaril^®^ Forte, containing 1,000 mg of citicoline sodium salt) added to conventional treatment (treatment group - TG). Each subject underwent a clinical evaluation and neurophysiological assessment using TMS, pretretament and posttreatment.

**Results:**

A total of thirty participants (mean [SD] age, 68.1 [9.6] years; 11 women [37%]) completed the study. We did not observe significant changes in clinical scores after CDP-choline treatment (all *p* > 0.05), but we observed a significant improvement in short-interval intracortical inhibition (SAI) (*p* = 0.003) in the TG group compared to the CG group.

**Conclusions:**

The eight-week treatment with citicoline after acute ischemic stroke may restore intracortical excitability measures, which partially depends on cholinergic transmission. This study extends current knowledge of the application of citicoline in acute ischemic stroke.

## Introduction

Ischemic stroke is one of the most devastating diseases (often involving severe physical damage) with more than 50% of stroke survivors presenting persistent disability, and about 30% still living with partial dependency on daily living activities 6 months after stroke (1, 2). Another post-stroke complication consisted of a series of syndromes from mild cognitive impairment to dementia, with an increased risk by at least five to eight times (3). For these reasons, stroke has been classed as a medical emergency and it is important to find new protective therapies beyond the acute phase (1, 4). Within the last few years, recent research on animal models of ischemic stroke supports the idea that pharmacological treatments potentially enhancing intrinsic brain plasticity could modulate acute brain damage, improving functional recovery, even when they are administered several h after the onset (5–7). In this scenario, it has been demonstrated that citicoline could provide neurovascular protection and repair effects in patients suffering from stroke (8).

Citicoline (or CDP-choline) is physiologically present in all human cells, and it acts as a neuroprotective compound as well as an intermediate in membrane phosphatide biosynthesis (9). In human, citicoline is degraded to cytidine and choline through hydrolysis and dephosphorylation. Thus, cytidine and choline represent substrates for the synthesis of phosphatidylcholine and CDP-choline in neurons (10, 11).

Up to now, citicoline has been widely studied in patients with various neurological conditions (12, 13). Considering patients suffering from stroke, contrasting findings were reported, with different studies that supported a beneficial effect of citicoline on clinical measures (13) but at least one sizeable multicentre study did not (4). However, no study has investigated potential beneficial effects on brain neurotransmitters circuits to further corroborate citicoline efficacy.

One of the latest approaches which may help to understand the neurophysiology of acute ischemic stroke is transcranial magnetic stimulation (TMS), which allows to indirectly assess neuronal circuits by applying paired-pulse TMS protocols (14).

In particular, short-afferent latency inhibition (SAI) allows to indirectly assess cholinergic circuits, while short-interval intracortical inhibition (SICI) and intracortical facilitation (ICF) protocols assess GABAergic and glutamatergic neurotransmission, respectively.

Overall, TMS is safe and well-tolerated and can be exploited as a non-invasive tool that can evaluate *in vivo* the cortical excitability, the propension to undergo neural plastic phenomena, and the underlying transmission pathways. In particular, patients suffering from stroke are characterized by lower motor excitability in the affected hemisphere (15) with also an interhemispheric imbalance in motor primary areas of both hemispheres, resulting in an asymmetric inhibition from the unaffected hemisphere (16). The objective of the present study was to evaluate the effects of citicoline on neuronal circuits, evaluated by TMS. To this, we carried out a pilot, randomized, single-blind clinical trial in a cohort of patients with acute ischemic stroke.

## Methods

### Participants

A total of thirty patients with acute ischemic stroke were recruited from the Stroke Unit, ASST Spedali Civili Hospital, Brescia, Italy within 36 h after the onset of symptoms and entered the study.

For each patient, past medical history was carefully recorded, and each patient underwent clinical and neurological examination, as well as brain structural imaging.

The inclusion criteria consisted of patients older than 60 years old and with a National Institutes of Health Stroke Scale (NIHSS) <14 and not treated with reperfusion treatments (thrombolysis with intravenous recombinant tissue plasminogen activator (rTPA) and/or mechanical thrombectomy) for known contraindication (time window and/or clinical/anamnestic factors that increased the hemorrhagic risk) (4, 17). We excluded cases with severe head trauma in the past, history of seizures, ischemic or hemorrhagic stroke, intracranial expansive process, pacemaker, metal implants in the head/neck region, and severe comorbidity *(*i.e., cancer in the past 5 years, non-controlled hypertension).

Full written informed consent was obtained from all participants according to the Declaration of Helsinki. The study protocol was approved by the local ethics committee (Brescia Hospital, #NP2982).

### Study Design

Patients were randomly assigned to two groups with a 1:1 ratio; the control group (CG) received conventional treatment (antiplatelet or anticoagulant drugs, statin, antihypertensive therapy according to current guidelines), and the treatment group (TG) received CDP-choline (Rischiaril^®^ Forte, containing 1,000 mg of citicoline sodium salt) in addition to conventional treatment for 8 weeks.

At baseline (T0) and at 8-weeks follow-up (T1), each participant underwent a standardized assessment of neurological deficits and cognitive functions and a standardized TMS protocol.

Neurological deficits were evaluated using the NIHSS (17), a 15-item scale that measures the level of neurological impairment, and the modified Rankin score (mRs) (18), a measure of functional disability. A brief cognitive evaluation was performed with the Mini-Mental State Examination (MMSE) (19). TMS protocols were carried out as described below.

The primary endpoint was defined as a significant change from baseline in neurophysiological measures, evaluated indirectly with TMS. The secondary endpoint was defined as changes from baseline in clinical assessment.

The examiners were blinded regarding the type of treatment when performing clinical ratings (EP, NG, AC, ID, RS, and MG) and TMS protocols (VC).

MM was responsible for random allocation sequences, participants' enrolment, and participants' assignation to specific interventions. Computer-assisted randomization was applied to allocate subjects into groups.

### Transcranial Magnetic Stimulation Assessment

A TMS figure-of-eight coil (each loop diameter 70 mm – D70^2^ coil) connected to a monophasic Magstim Bistim^2^ system (Magstim Company, Oxford, UK) was employed for all TMS paradigms, as previously reported (20). Patients were stimulated on the ischemic lesion side. Electromyographic (EMG) recordings were performed from the contralateral first dorsal interosseous muscle using 9 mm diameter Ag-AgCl surface-cup electrodes. The active electrode was placed over the muscle belly and the reference electrode over the metacarpophalangeal joint of the index finger. Responses were amplified and filtered at 20 and 2 kHz with a sampling rate of 5 kHz. The TMS coil was held tangentially over the scalp region corresponding to the primary hand motor area contralateral to the target muscle, with the coil handle pointed 45° posteriorly and laterally to the sagittal plane.

Resting motor threshold (RMT) was determined as the minimum intensity of the stimulator required to elicit motor evoked potentials (MEPs) with a 50 μV amplitude in 50% of 10 consecutive trials, recorded during full muscle relaxation (21).

SICI-ICF and SAI were studied using a paired-pulse technique, employing a conditioning-test design. For all paradigms, the test stimulus (TS) was adjusted to evoke an MEP of approximately 1 mV amplitude.

For SICI and ICF, the conditioning stimulus (CS) was adjusted at 70% of the RMT, employing multiple interstimulus intervals (ISIs), including 1, 2, and 3 ms for SICI and 7, 10, and 15 ms for ICF (22, 23). SAI was evaluated employing a CS of single pulses (200 μs) of electrical stimulation delivered to the right median nerve at the wrist, using a bipolar electrode with the cathode positioned proximally, at an intensity sufficient to evoke a visible twitch of the thenar muscles (24). Different ISIs were implemented (0 and +4), which were fixed relative to the N20 component latency of the somatosensory evoked potential of the median nerve (24).

For each ISI and for each protocol, ten different paired CS-TS stimuli and fourteen control TS stimuli were delivered to all participants in a pseudo-randomized sequence, with an inter-trial interval of 5 secs (±10%).

The conditioned MEP amplitude, evoked after delivering a paired CS-TS stimulus, was expressed as a percentage of the average control MEP amplitude. Average values for SICI (1, 2, and 3 ms ISI), ICF (7, 10, and 15 ms ISI), and SAI (0 and +4 ms ISI) were used for analysis.

Stimulation protocols were conducted in a randomized order. Audio-visual feedback was provided to ensure muscle relaxation during the entire experiment and trials were discarded if EMG activity exceeded 100 μV in the 250 ms prior to TMS stimulus delivery. Less than 5% of trials were discarded for each protocol. All of the participants were capable of following instructions and reaching complete muscle relaxation; if, however, the data were corrupted by patient movement, the protocol was restarted and the initial recording was rejected.

### Statistical Analyses

Continuous and categorical variables are reported as mean (± standard deviation) and percentage (number), respectively. Demographic and clinical data were assessed using the Mann-Whitney U test for continuous variables and Chi-square test for categorical variables, as appropriate. To assess the effect of CDP-choline treatment on neurophysiological or clinical measures over time, we used a two-way repeated-measures ANOVA with TIME (T0 and T1) and TREATMENT (CG and TG groups) as within-subjects factors. Statistical analyses were performed using SPSS version 21 (SPSS, Inc., Chicago, IL, USA).

### Data Availability

All study data, including study design, protocol, statistical analysis plan, and results, are available from the corresponding author upon reasonable request.

## Results

### Participants

A total of thirty-three participants with acute ischemic stroke entered the study; three patients were excluded from analyses due to meeting the exclusion criteria (*n* = 2 with unexcitable motor cortex and *n* = 1 carrying a pacemaker). A final count of 30 patients (16 with the right-sided lesions, 14 with left-sided lesions) was considered in the present study and was randomized. The two groups did not differ in demographic and clinical characteristics at baseline as well as at follow-up. The location of the stroke and the subtype classification [according to TOAST criteria (25)] did not differ among groups (see Table 1).

**Table 1:** Demographic and clinical characteristics of included patients.

**Variable**	**All patients**	**TG**	**CG**	***P*-value**°****
Patients, *n*	30	15	15	-
Age, years	68.1 ± 9.6	69.1 ± 8.4	67.1 ± 10.8	0.68
Sex, % women (*n*)	36.7 (11)	53.3 (8)	20.0 (3)	0.06^∧^
Education, years	8.9 ± 3.6	9.5 ± 4.3	8.3 ± 2.8	0.59
BMI	25.8 ± 3.8	26.1 ± 4.2	25.4 ± 3.3	0.92
Side of stroke, % left (*n*)	46.7 (14)	40.0 (6)	53.3 (8)	0.46^∧^
Stroke location^#^, *n*	10/3/7/3/7	7/2/4/0/2	3/1/3/3/5	0.17^∧^
TOAST classification^§^, n	2/14/5/9	2/7/3/3	0/7/2/6	0.36^∧^
Diabetes, % (*n*)	16.7 (5)	6.7 (1)	26.7 (4)	0.14^∧^
Hypertension, % (*n*)	53.3 (16)	53.3 (8)	53.3 (8)	1.00^∧^
Hypercholesterolemia, % (*n*)	50.0 (15)	46.7 (7)	53.3 (8)	0.72^∧^
Cardiopathy, % (*n*)	30.0 (9)	26.7 (4)	33.3 (5)	0.70^∧^
Atherosclerosis, % (*n*)	50.0 (15)	46.7 (7)	53.3 (8)	0.72^∧^
Smoke, % (*n*)	53.3 (16)	40.0 (6)	66.7 (10)	0.33^∧^

*Results are expressed as mean ± standard deviation, unless otherwise specified. TG, treatment group; CG, control group; BMI, Body mass index; MCA, middle cerebral artery. ^#^frontal/Internal capsule/MCA territory/temporal/basal ganglia; ^§^large-artery atherosclerosis/cardioembolism/stroke of undetermined etiology/small-vessel occlusion; °Mann-Whitney U-test unless otherwise specified; ^∧^Pearson Chi-Square test*.

### Effect of CDP-Choline Treatment on Neurophysiological and Clinical Assessment

Baseline and follow-up clinical and neurophysiological scores are reported in Table 2. No statistically significant differences in clinical measures (at baseline, follow-up, or at the TIME × TREATMENT interaction) were evident.

**Table 2:** Clinical and neurophysiological parameters of included patients before and after CDP-choline or standard treatment.

**Variable**	**CDP-choline treatment**		**Standard treatment**	
	**T0**	**T1**	**T0**	**T1**
***Clinical assessment***				
NIHSS	4.6 ± 6.2	0.9 ± 1.5	5.1 ± 4.2	0.9 ± 1.1
mRS	1.3 ± 1.6	0.5 ± 0.7	1.5 ± 1.6	0.7 ± 0.8
MMSE	28.4 ± 1.9	28.3 ± 2.5	25.7 ± 5.6	26.4 ± 4.9
***TMS***				
Mean SICI (1, 2, 3 ms)	0.52 ± 0.30	0.41 ± 0.23	0.45 ± 0.20	0.44 ± 0.20
Mean ICF (7, 10, 15 ms)	1.21 ± 0.20	1.27 ± 0.12	1.21 ± 0.22	1.17 ± 0.16
Mean SAI (0, +4 ms)	0.81 ± 0.21	0.51 ± 0.18^∧*^	0.79 ± 0.17	0.76 ± 0.26

Results are expressed as mean ± standard deviation. NIHSS, National Institutes of Health Stroke Scale; mRS, modified Rankin score; MMSE, Mini-Mental State Examination; SICI, short interval intracortical inhibition; ICF, intracortical facilitation; SAI, short-afferent latency inhibition. ^∧^significant difference compared to Standard Treatment; *significant difference compared to T0 (General Linear Model for repeated measures).

**Figure 1:**
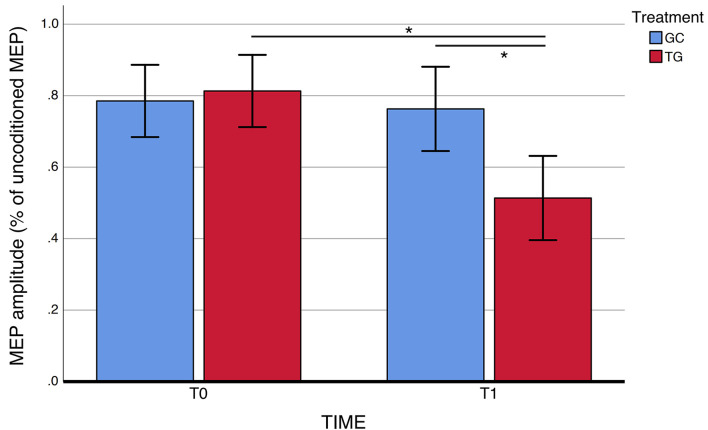
SAI measures before and after exposure to CDP-choline treatment. SAI, short-latency afferent inhibition. Error bars represent standard errors. *Significant difference.

For SAI, there was a statistically significant TIME × TREATMENT interaction at the repeated measures ANOVA (*F* = 9.94, *p* = 0.004, partial η^2^ = 0.29), with a significantly restored cholinergic transmission at T1 (average.51 ±0.18) compared to T0 (average.81 ±0.21) in the CDP-choline treatment group (Figure 1 and Table 2). No statistically significant TIME × TREATMENT differences were observed for SICI (*F*= 1.56, *p* = 0.223, partial η^2^ = 0.03) and ICF (*F* = 3.75, *p* = 0.063, partial η^2^ = 0.04).

## Discussion

The treatment of acute ischemic stroke remains a page still largely to be written, given that, unfortunately, it continues to be a fearful disease, especially for its disabling results.

Stroke has been classed as a medical emergency and it is important to find new effective protective therapies.

In this randomized, single-blind pilot study, we demonstrated the beneficial effect of citicoline in restoring SAI in patients with acute ischemic stroke. SAI is a marker of sensorimotor integration which partially and indirectly reflects cholinergic inhibition mediated by GABA_A_ receptors (26, 27). Literature data on the relationship between stroke and the cholinergic system reported an impaired cholinergic activity (choline acetyltransferase and acetylcholinesterase) in patients suffering from stroke (28). Moreover, SAI has been shown to correlate with the degree of motor impairment after stroke (29). Interestingly, in our study, the impairment of SAI persisted after 8 weeks in patients in the control group. Citicoline has been proved to modulate different neurotransmitter pathways in clinical studies as well as in animal models of disease (13, 30, 31). Recently, the cholinergic system and the extended hippocampal network (primarily involving the nucleus basalis of Meynert) have been identified as the main players in cognitive recovery after stroke, supporting the idea that targeted therapeutic strategies could enhance spontaneous mechanisms of recovery (32, 33). Previous randomized clinical trials on citicoline in stroke have reported a mixed effect (34), with specific clinical factors (patients >70 years of age, moderate stroke severity, utilization of recanalization treatments (i.e., rt-PA thrombolysis and/or mechanical thrombectomy) potentially affecting clinical efficacy of citicoline in the acute phase of ischemic stroke (4). Moreover, clinical trials on the cholinergic modulation in stroke (using Donepezil) have reported inconclusive results (35–38). Interestingly, TMS in vascular cognitive impairment demonstrated increased cortical excitability and synaptic plasticity as adaptative responses potentially related to disease progression (39). Thus, TMS could be used to forecast cognitive deterioration in subjects “at-risk” for dementia (chronic vascular encephalopathy, leukoaraiosis, etc.) (39), in light of disease-modifying/neuromodulatory treatments. From this perspective, TMS assessment (considering the SAI protocol) may represent an effective and feasible tool to detect those patients with an established cholinergic deficit that could benefit more from a targeted treatment for cholinergic restoration, as already studied in vascular cognitive impairment (39–42). Thus, for the first time, the present study demonstrated *in vivo* modulation of the cholinergic system by the utilization of citicoline in patients with ischemic stroke, paving the way for a personalized medicine approach to potentiate the clinical recovery after ischemic stroke.

Therefore, TMS can be exploited, as in this case, to evaluate the response to specific pharmacological treatments in the attempt to not only identify new therapeutic targets but also to predict cognitive deterioration caused by stroke. The role of TMS in cerebrovascular diseases is catching on cortical excitability, plasticity, and connectivity, also providing new clues on the pathophysiology of the impairment with a translational perspective toward novel treatments for these patients (27).

We acknowledge that the present pilot study entails some limitations. First, the group sample is limited, even though well characterized. Moreover, correlations between neurophysiological and clinical variables should be considered in larger samples (also considering the potential modulating effect of recanalization treatments) to corroborate the present findings. Taking into account these caveats, the present approach for the evaluation and modulation of the cholinergic system in ischemic stroke should warrant further studies.

## Data Availability Statement

The raw data supporting the conclusions of this article will be made available by the authors, without undue reservation.

## Ethics Statement

The studies involving human participants were reviewed and approved by Comitato Etico, ASST Spedali Civili, Brescia. The patients/participants provided their written informed consent to participate in this study.

## Author Contributions

EP: conception and design of the work, acquisition of the data, statistical analysis, and draft of the manuscript. VC: acquisition of the data, statistical analysis, and draft of the manuscript. AB: conception and design of the work, acquisition of the data, statistical analysis, and revision of the manuscript. NG, VV, ID, MG, RS, and AC: acquisition of the data and revision of the manuscript. AP: critical revision of the manuscript for intellectual content. BB: conception and design of the work and draft of the manuscript. MM: conception and design of the work and revision of the manuscript. All authors contributed to the article and approved the submitted version.

## Funding

AB was partially supported by the Airalzh-AGYR2020 and by Fondazione Cariplo, grant n° 2021-1516.

## Conflict of Interest

The authors declare that the research was conducted in the absence of any commercial or financial relationships that could be construed as a potential conflict of interest.

## Publisher's Note

All claims expressed in this article are solely those of the authors and do not necessarily represent those of their affiliated organizations, or those of the publisher, the editors and the reviewers. Any product that may be evaluated in this article, or claim that may be made by its manufacturer, is not guaranteed or endorsed by the publisher.
